# Taraxacum alleviates ulcerative colitis, accompanied by the modulation of gut microbiota and restoration of intestinal barrier integrity

**DOI:** 10.3389/fcimb.2026.1778487

**Published:** 2026-03-27

**Authors:** Chunsheng Fu, Zheyu Chen, Chensen Xu, Huilian Hua, Jun Ye

**Affiliations:** 1 Taizhou People’s Hospital, Nanjing Medical University, Taizhou, Jiangsu, China; 2 Medical Department, Yangzhou University School of Medicine, Yangzhou, Jiangsu, China

**Keywords:** antioxidation, gut flora, immune function, taraxacum, ulcerative colitis

## Abstract

**Background:**

Previous studies have shown that *Taraxacum*(TM) possesses strong antioxidant, anti-inflammatory, and antibacterial activities, but the mechanism regarding how TM attenuates IBD requires further exploration. This study evaluated the therapeutic effects of TM (0.15, 0.75, and 1.5 g/kg) on ulcerative colitis induced by Dextran Sulfate Sodium (DSS) in mice.

**Results:**

After 14 days of treatment following colitis induction, the herbal extract alleviated body weight loss and pathological abnormalities in the mice. In comparison to the DSS group, the 1.5 g/kg The TM therapy group had markedly elevated levels of colonic T-SOD, T-AOC, and GSH-Px (P < 0.05), alongside a substantial decrease in MDA content (P < 0.05) and inflammatory cytokines (IL-2, IL-6, IFN-g, TNF-a) (P < 0.05). Furthermore, TM markedly elevated the concentrations of volatile fatty acids (acetate, propionate, isobutyrate, and butyrate) in the cecal contents (P < 0.05). The 0.75 and 1.5 g/kg TM groups also elevated the expression of Claudin-1, Occludin, and ZO-1 (P < 0.05), whereas the DSS group showed reduced expression of these mucosal barrier proteins. Concerning gut microbiota, at the Phylum level, the relative abundance of *Firmicutes* was markedly elevated in the 1.5 g/kg TM groups (P < 0.05), *Bacteroidetes* exhibited a considerable reduction (P < 0.05); TM alleviated colitis concurrent with the restoration of beneficial *Muribaculaceae* and reduction of harmful *Desulfovibrio*. Notably, *Desulfovibrio* was inhibited more effectively than in 5-ASA, a change associated with intestinal homeostasis.

**Conclusion:**

TM alleviated colitis is correlated with enhancing antioxidant capacity, reducing inflammation, restoringbarrier integrity, and modulating the gut microbiota.

## 1 Introduction

Inflammatory bowel disease (IBD) is an immune-mediated condition of the gastrointestinal system marked by persistent and recurring inflammation, it primarily encompasses ulcerative colitis (UC) and Crohn’s disease (CD) (Baumgart and Le Berre, 2021). CD lesions predominantly manifest in the terminal ileum and may include the entire gastrointestinal system, whereas UC lesions are usually confined to the colon and mainly present as erosive lesions of the colonic mucosa (Zhang et al., 2014). The pathogenesis of UC was generally considered to be multifactorial, involving environmental factors, genetic susceptibility, and other contributors that trigger immune responses, resulting in damage to the intestinal epithelial barrier and inflammatory cascades that ultimately led to chronic inflammation (Lechuga and Ivanov, 2017). Clinically, Ulcerative colitis presented with abdominal pain, diarrhea, hematochezia, weight loss, and additional symptoms, with a prolonged and relapsing course accompanied by various complications such as intestinal strictures and abscesses (Du and Ha, 2020). Data from epidemiological studies show that the incidence and prevalence of UC continue to be significant in developed nations, including those in Europe and the United States, and trend was experiencing a significant rise in developing countries, marked by an annual growth rate of 14.9% (Ng et al., 2017; Ungaro et al., 2017). Studies have shown that the hospitalization rate of UC in China rose from 6.24 per 100,000 people in 2013 to 8.29 per 100,000 in 2018 (compound annual growth rate = 5.73%). The economic burden related to medical resource utilization for UC ranges from 1,200 to 1,500 USD, indicating that the treatment of UC poses a substantial challenge to global healthcare systems (He et al., 2022).

Presently, the pharmacological agents used in the therapeutic management of ulcerative colitis mostly consist of 5-aminosalicylic acid, corticosteroids, and immunosuppressants. However, these drugs often have certain adverse effects and limitations in clinical efficacy (Berends et al., 2019; Kucharzik et al., 2020). For example, aminosalicylic acid analogues cause loss of appetite, nausea and vomiting, and may have adverse effects such as autoimmune haemolysis and granulocytopenia (Talaei et al., 2013). Corticosteroids cause osteoporosis, cardiovascular disease, impaired immune function, growth suppression and peptic ulcers (Rice et al., 2017). Immunosuppressants, on the other hand, are cytotoxic and their long-term use may lead to liver and kidney impairment and bone marrow haematopoietic dysfunction (Nielsen et al., 2019). Consequently, there is an imperative need to devise innovative therapies that are effective, safe, and natural.

TM is rich in various active compounds, including monoterpenes, flavonoids, phenolic compounds, saponins, vitamins, carbohydrates, fatty acids, and proteins (Sharifi-Rad et al., 2018). Comprehensive research, conducted in both laboratory environments and clinical trials, indicates that TM exhibits considerable anti-inflammatory and antioxidant (Schütz et al., 2006; Martinez et al., 2015; Sharifi-Rad et al., 2018). The oral administration of TM extract in the gastrointestinal tract has demonstrated efficacy in mitigating gastritis and oxidative damage in rat models by decreasing malondialdehyde levels and pro-inflammatory cytokines, such as TNF-α (Yang et al., 2017), while simultaneously enhancing gastric motility (Jin et al., 2011). Although TM is acknowledged for its protective properties on the stomach, its effectiveness in addressing ulcerative colitis remains ambiguous. A recent study reveals that many plant extracts mitigate intestinal inflammation by influencing the gut microbiota and safeguarding the mucosal barrier (Pang et al., 2025); nevertheless, it remains unclear if TM achieves its anti-colitis effects through analogous microbiome-related processes. Several critical questions persist concerning how TM intervention affects gut microbiota composition, alters the balance between beneficial and harmful bacteria, and impacts subsequent metabolic pathways, including the synthesis of short-chain fatty acids (SCFAs) during ulcerative colitis (UC). Thus, the present study utilized a DSS-induced mouse model to evaluate the protective role of TM and identify the key mechanisms involved.

## 2 Materials and methods

### 2.1 Materials and reagents

Dextran Sulfate Sodium Salt (DSS) and 5-aminosalicylic acid (5-ASA) were acquired from MP Biomedical (USA); 4% paraformaldehyde fixative was sourced from Wuhan Servicebio Biotechnology (Wuhan, China) Company Limited; the Mouse interleukin (IL)-2 ELISA kit, Mouse IL-6 ELISA kit, Mouse tumor necrosis factor (TNF)-α ELISA kit, Mouse interferon (IFN)-γ ELISA kit, total antioxidant capacity (T-AOC), total superoxide dismutase (T-SOD), glutathione peroxidase (GSH-Px), and malondialdehyde (MDA) were obtained from Nanjing JianCheng Bio-technology Co. Ltd (Nanjing, China).

### 2.2 Major instruments and equipment

METTLER XS204 electronic balance, Mettler Toledo; EPPENDORF 5424 benchtop centrifuge, Eppendorf AG; Multiskan MK3 enzyme labeller, Thermo Fisher Scientific.

### 2.3 Experimental animals

Experimental subjects: SPF grade female C57BL/6 mice, 5 weeks old, acquired from Jicui Yaokang Biotechnology Co., Ltd. The mice were kept in an environment with a temperature range of 24 to 26°C and humidity levels between 50% and 60%, following a 12-hour light/dark cycle. All techniques of this experiment received approval from the Ethical Review Committee for Laboratory Animal Welfare of Hanjiang Biotechnology Co., Ltd (HJSW-23052310). All animal trials were performed in compliance with ARRIVE guidelines.

### 2.4 Experimental methods

#### 2.4.1 Preparation of DSS solution

DSS was dissolved in sterilized distilled water to create a 3% (w/w) solution, which was then sterilized by using a 0.22 μm filter membrane.

#### 2.4.2 Preparation of aqueous extract of Taraxacum

1 kilogram of Taraxacum was combined with 10 liters of clean water, and then decocted for 90 minutes. The solution was obtained by decocting the liquid twice and filtering it twice using filter paper. The solution was introduced into the rotary evaporator, maintained at 60°C, concentrated by distillation, transferred onto a petri dish, sealed with aluminum foil, and then lyophilized. Three days later, the lyophilized medication was processed in a grinder to produce a brownish powder, with a final extraction yield of 16.3% (w/w), which was thereafter packed and stored. The extraction process strictly adheres to standardized procedures, the primary active components of the dandelion extract obtained through this method such as chlorogenic acid, cichoric acid, and flavonoids had been thoroughly characterized in previous high-quality literature (Qu et al., 2022).

#### 2.4.3 Animal grouping and modelling

Forty-eight C57BL/6 mice were chosen and maintained in a controlled setting with commercial rodent diet and sterile water for seven days. According to the principle of similar body mass, they were categorized into the following six groups according to a completely randomized design: blank control group (Control), model group (DSS), positive drug group (5-ASA), Taraxacum low dose group (0.15 g/kg TM), Taraxacum medium dose (0.75 g/kg TM) group, Taraxacum high dose group (1.5 g/kg TM), 8 animals in each group, the doses of TM selected were based on previous reports (Davaatseren et al., 2013; Yan et al., 2023). Control group: given normal drinking water, while using sterile water gavage for 21 d; DSS model group: 3% concentration of DSS solution, free to drink, while using sterile water gavage for 21 d; 5-ASA: started on the 7th day of 3% DSS solution drinking with 5-ASA: 14-d gavage with different doses of Taraxacum (low: 0.15 g/kg/d, medium: 0.75 g/kg/d, and high: 1.5 g/kg/d) starting from the 7th day of drinking 3% DSS solution, and the mice were sacrificed on the 21st day.

#### 2.4.4 General condition of mice and disease activity index scores

The general conditions of the mice in each group, such as body weight, mental status, body surface characteristics, fecal characteristics, etc., were observed and recorded daily, and fecal occult blood tests were performed. The formula for calculating the DAI score of mice in each group was DAI = (body mass loss rate + stool trait score + blood in stool score)/3. Body mass loss rate = (daily body mass - initial body mass)/initial body mass × 100%.

#### 2.4.5 Sample collection

After the last administration, mice were fasted for 12 h, anesthetized with 3% pentobarbital sodium, blood was collected from the eye socket, placed in a clean test tube, centrifuged at 4°C, 4–000 rpm/min for 12 min, and the serum were separated. At the experimental endpoint, mice were euthanized by cervical dislocation in accordance with institutional guidelines. Then, the colon tissue was cut 1 cm upward from the anus and fixed in 4% paraformaldehyde solution for 0.5 cm, and the rest of the colon tissue was used for the subsequent detection of related indexes.

#### 2.4.6 HE staining

Colon tissues fixed in 4% paraformaldehyde were subjected to dehydration, paraffin embedding, sectioning, and drying. Subsequently, they were deparaffinized using xylene. Rehydrated in a graded alcohol series, followed by hematoxylin and eosin staining, dehydrated with gradient alcohol, cleared with xylene, and mounted with neutral gum. Various fields of view from each segment were chosen to examine the histological alterations of the colon under a light microscope.

#### 2.4.7 Antioxidant enzyme assay

T-AOC, T-SOD, GSH-Px and MDA levels in mouse colon were determined using commercial kits and strictly following the kit instructions.

#### 2.4.8 Cytokine determination

IL-2, IL-6, TNF-α, and IFN-γ levels were quantified in mouse colon tissue using ELISA, with the experimental procedures conducted meticulously in accordance with the kit’s instructions.

#### 2.4.9 Western blot for tight junction proteins detection

Colonic tissues were homogenized using RIPA lysis buffer (Beyotime, China). The lysates were incubated on ice for 15 minutes and subsequently centrifuged at 12,000 rpm for 15 minutes at 4°C. Then, the supernatants were gathered, and the protein concentration was measured utilizing a BCA Protein Assay Kit (Beyotime, China). After SDS-PAGE fractionation of 30 μg protein per sample, the proteins were electroblotted onto polyvinylidene difluoride (PVDF) membranes (Millipore, USA). Membranes were first blocked using 5% non-fat dry milk in TBST for 60 min at room temperature. Subsequently, incubation with primary antibodies targeting Claudin-1 (Abclonal, A2196, China), Occludin-1 (Abclonal, A2601, China), ZO-1 (Abclonal, A0659, China) were performed overnight at 4°C. Subsequent to the washing procedures, the membranes were incubated with HRP-conjugated secondary antibodies (Biosharp, China) for 1 hour at ambient temperature. Protein signals were identified with an improved chemiluminescence reagent (ECL, Millipore, USA), and band intensities were measured utilizing ImageJ software (NIH, USA). β-actin served as the internal loading control.

#### 2.4.10 Determination of volatile fatty acids content

Volatile fatty acids (VFAs) profiling was conducted with an Agilent GC7890 Network System. Before analysis, 1 g of cecal digesta was acidified with 6% phosphoric acid (1:5, w/v) and subsequently injected into the column (30 mm × 0.25 mm × 0.25 mm). Column (HP-FFAP, Agilent Technologies) for flame ionization.

#### 2.4.11 Analysis of the cecum microbiota

This research used the EZNA DNA extraction kit to isolate DNA from mouse cecum contents. The V3–V4 hypervariable region of the bacterial 16S rRNA gene was amplified with the particular primer pair (338F: 5’-ACTCCTACGGGGAGGCAGCAG-3’; 806R: 5’-GGACTACHVGGGTWTCTAAT-3’). Sequencing was conducted by Shanghai Meiji Biomedical Technology Co.

#### 2.4.12 Statistical analyses

Statistical analysis was conducted using SPSS version 25.0. Group differences were evaluated using one-way ANOVA, followed by Tukey’s technique. Comparisons were used to examine the significant differences among the data. GraphPad Prism 8.0 was used for graphing, with P < 0.05 denoting statistically significant differences.

## 3 Results

### 3.1 The effects of Taraxacum on body weight changes, disease activity index scores and colon tissues in UC mice

**Figure 1:**
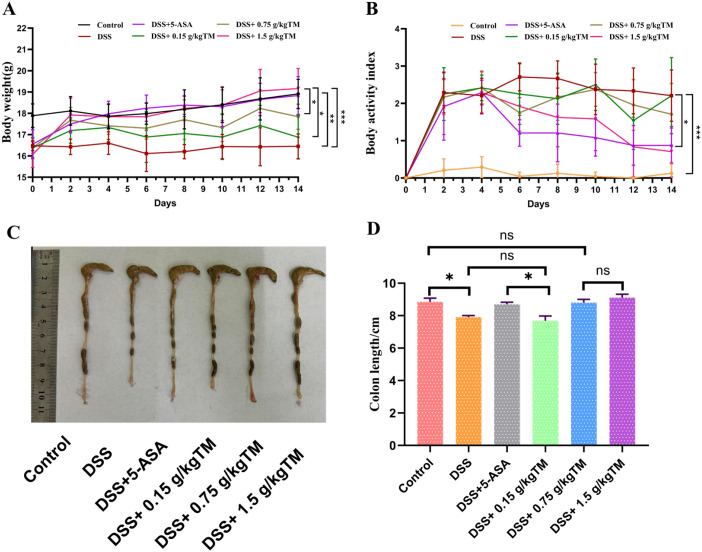
The effects of Taraxacum on body weight changes, disease activity index scores and colon tissues in UC mice. *P < 0.05; **P < 0.01; ***P < 0.001. **(A)** Body weight changes during the 14-day experimental period; **(B)** Disease activity index (DAI) scores; **(C)** Representative visual images of colon tissues from each group; **(D)** Statistical analysis of colon length. The value is the mean ± SEM. ns = not significant.

On the concluding day of the trial, mice administered DSS exhibited a substantial reduction in body weight and a rising trend in DAI scores relative to the Control group (*P* < 0.05; **Figure 1**). These symptoms were alleviated in the 5-ASA and TM groups. No significant difference in body weight was seen between the 5-ASA and 1.5 g/kg TM groups (*P* > 0.05).; however, mice in 1.5 g/kg TM groups displayed higher weight when compared with other TM groups (*P* < 0.05). Moreover, relative to the DSS group, the colon length of mice in the 0.75 and 1.5 g/kg groups was dramatically augmented (*P* < 0.05).

### 3.2 The effect of Taraxacum on the histopathological morphology of colon tissue in UC mice

**Figure 2:**
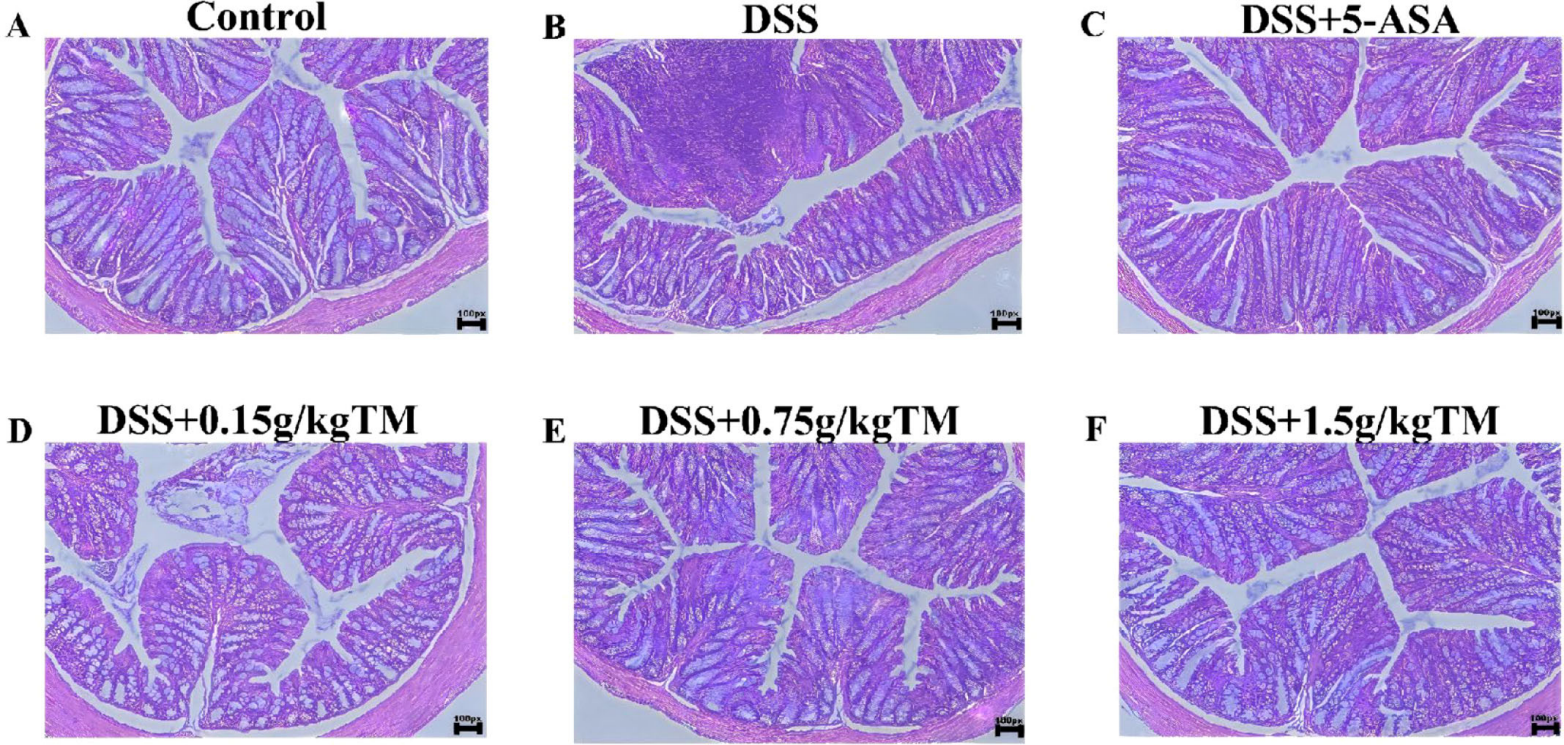
The effect of Taraxacum on the histopathological morphology of colon tissue in UC mice. **(A–F)** Representative H&E staining images of colon sections from the Control, DSS, DSS+5-ASA, DSS+0.15g/kgTM, DSS+0.75g/kgTM, and DSS+1.5g/kgTM groups, respectively.

Representative histological sections of the colon from six groups were shown: (A) Control group, (B) DSS group, (C) 5-ASA treated group, (D–F) different doses of TM treatment group (0.15 g/kg, 0.75 g/kg, 1.5 g/kg respectively) (**Figure 2**). The Control group exhibited intact epithelial architecture and well-organized crypts. The DSS-treated group demonstrated significant mucosal damage, marked by the destruction of crypt architecture and substantial infiltration of inflammatory cells. Treatment with TM markedly improved mucosal integrity and reduced inflammatory infiltration in a dose-dependent manner. The experimental compound with 0.15 g/kg TM also showed some shedding of villi and inflammatory infiltration, however, no significant abnormalities were observed in the other dosage groups.

### 3.3 The effect of Taraxacum on oxidative stress-related indicators in UC mice

**Figure 3:**
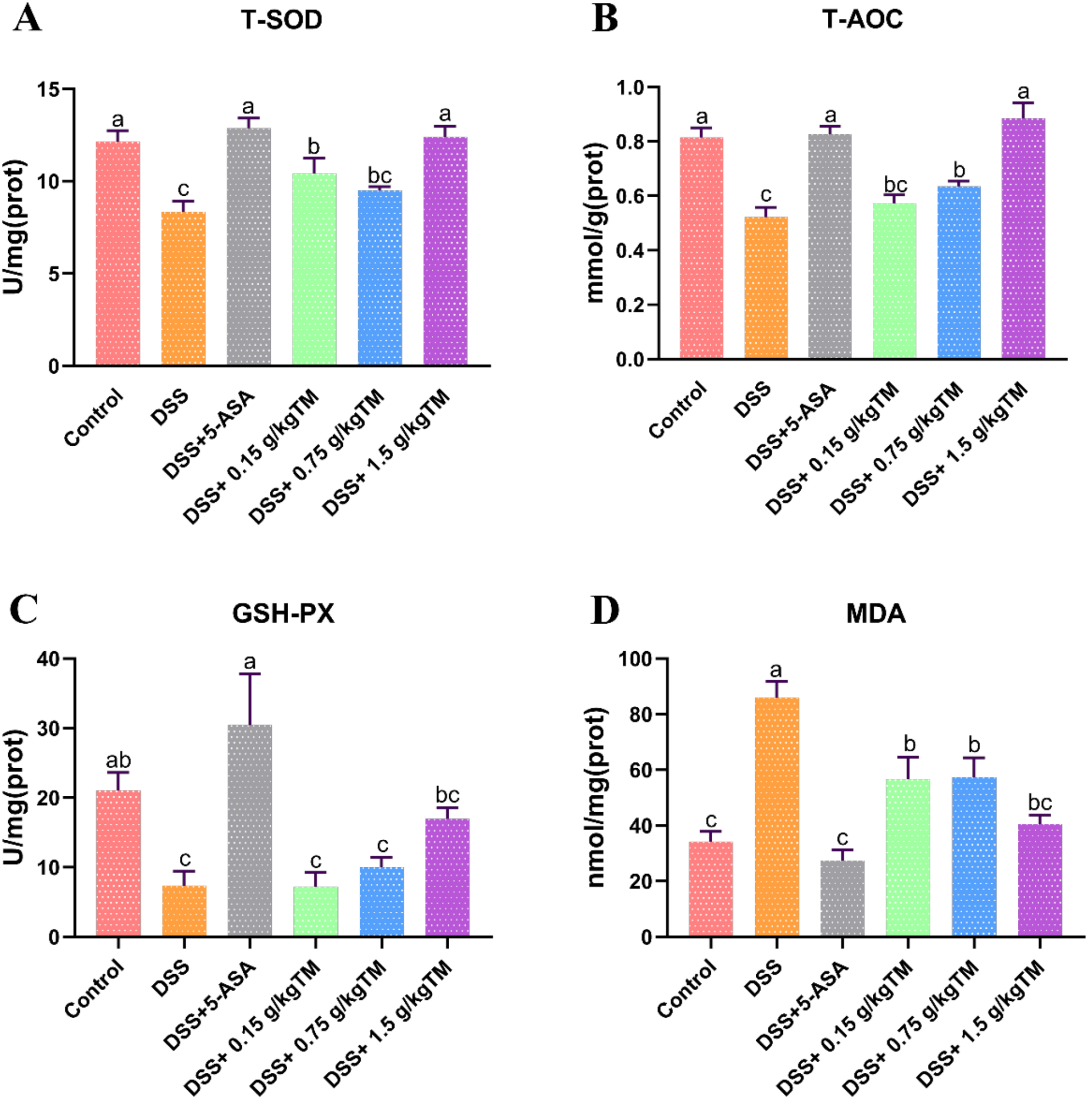
The effect of Taraxacum on oxidative stress-related indicators in UC mice. Bars assigned with different superscripts are significantly different (*P* < 0.05). **(A)** Total superoxide dismutase (T-SOD) activity; **(B)** Total antioxidant capacity (T-AOC); **(C)** Glutathione peroxidase (GSH-PX) activity; **(D)** Malondialdehyde (MDA) content. The value is the mean ± SEM.

Compared to the Control group, colonic T-SOD, T-AOC, and GSH-Px activities were markedly suppressed in DSS-challenged mice (**Figure 3**; *P* < 0.05), while those in the 5-ASA group were significantly increased (*P* < 0.05), indicating the therapeutic efficacy of 5-ASA. After TM treatment, the contents of T-SOD, T-AOC, and GSH-Px in the colon of DSS-treated mice were also markedly increased, showing a dose-dependent effect with increasing TM concentration. In comparison to the DSS group, levels of T-SOD, T-AOC, and GSH-Px were elevated in the 1.5 g/kg TM group (*P* < 0.05), respectively. In addition, in contrast to the DSS group, MDA levels in the Control, 5-ASA and 1.5 g/kg TM groups were significantly reduced (*P* < 0.05).

### 3.4 The effect of Taraxacum on the levels of inflammatory cytokines in UC mice

**Figure 4:**
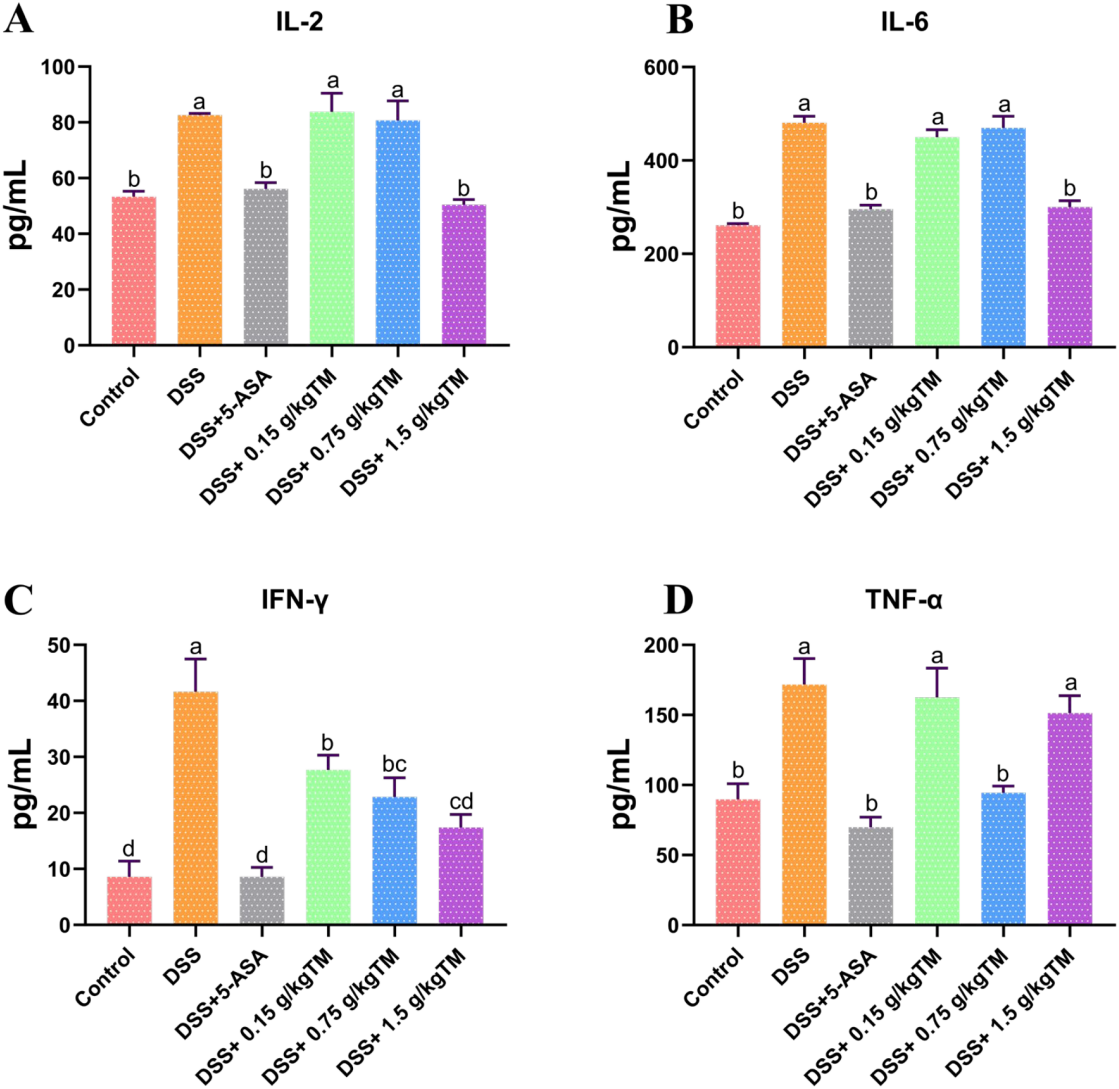
The effect of Taraxacum on the levels of inflammatory cytokines in UC mice. Bars assigned with different superscripts are significantly different (*P* < 0.05). **(A)** Interleukin-2 (IL-2) levels; **(B)** Interleukin-6 (IL-6) levels; **(C)** Interferon-γ (IFN-γ) levels; **(D)** Tumor necrosis factor-α (TNF-α) levels. The value is the mean ± SEM.

Exposure to DSS elicited a pronounced inflammatory response, as seen by the significant elevation of IL-2, IL-6, IFN-γ, and TNF-α levels, demonstrated a significant deviation compared to the Control group (**Figure 4**; *P* < 0.05), consequently confirming the effective development of the inflammatory phenotype. And 5-ASA exhibited a strong anti-inflammatory effect, moreover all TM-treated groups showed improvement in a dose-dependent manner. Notably, the levels of IL-2 and IL-6 in 1.5 g/kg group were significantly reduced (*P* < 0.05), achieving effects comparable to those of 5-ASA, and also significantly increased (*P* < 0.05) IFN-γ levels. Significantly, TNF-α had a unique response pattern, with the 0.75 g/kg dosage being the most effective for suppression. In contrast, neither the low (0.15 g/kg) nor the high (1.5 g/kg) doses resulted in significant reductions in TNF-α, with levels remaining comparable to the DSS model group (*P* > 0.05).

### 3.5 The effect of Taraxacum on the levels of tight junction proteins expression in UC mice

**Figure 5:**
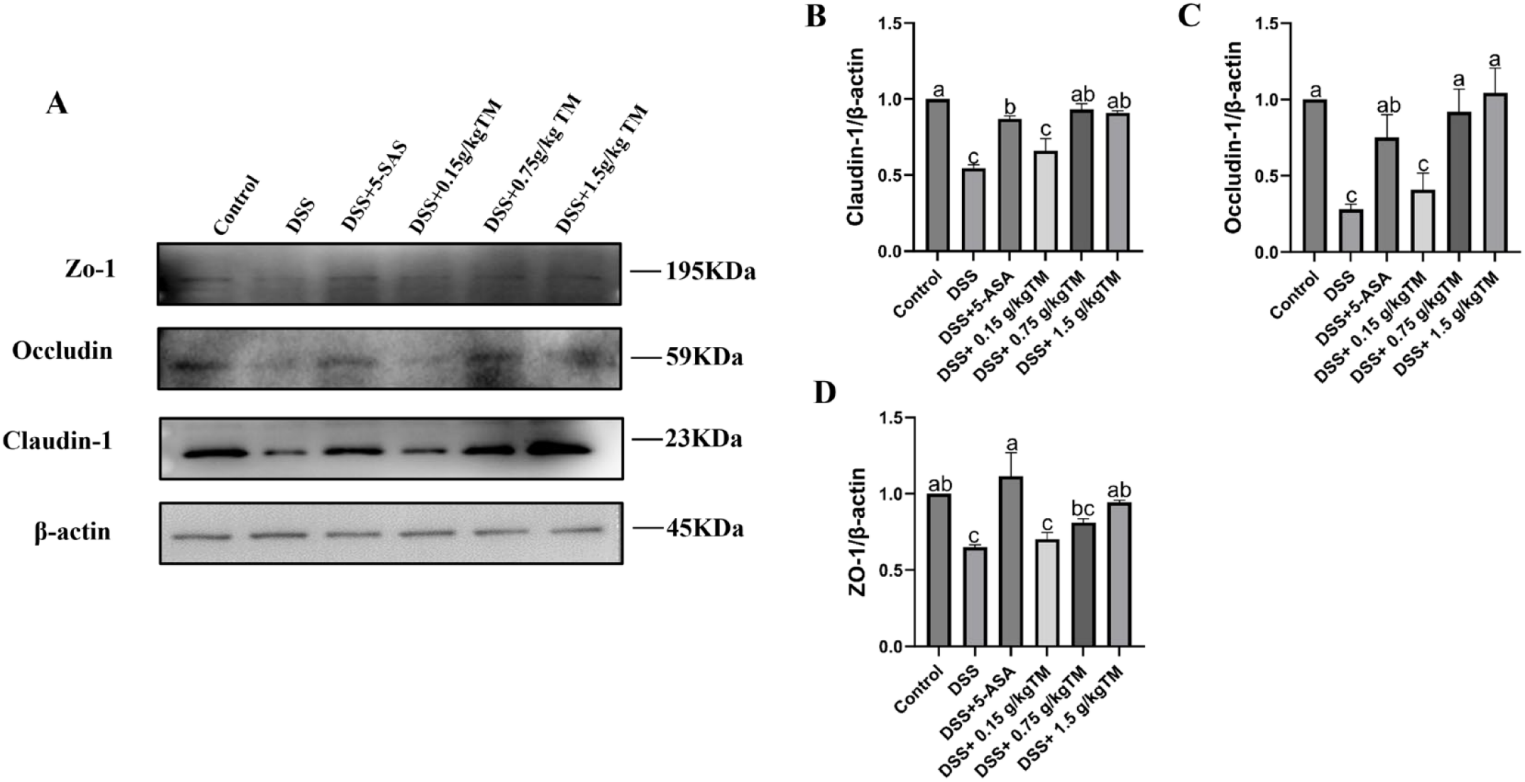
The effect of Taraxacum on tight junction proteins in UC mice. Bars assigned with different superscripts are significantly different (*P* < 0.05). **(A)** Representative Western Blot images of ZO-1, Occludin, and Claudin-1, with β-actin as the internal control; **(B–D)** Relative protein expression levels of Claudin-1, Occludin, and ZO-1 normalized to β-actin. The value is the mean ± SEM.

To further clarify the effects of TM intervention on epithelial tight junction integrity and innate immune signaling, we examined the expression of Claudin-1, Occludin-1, ZO-1 in colonic tissues by Western blot (**Figure 5**). As expected, DSS challenge markedly disrupted mucosal barrier proteins. The integrity of the mucosal barrier was notably disturbed, as indicated by the significant reduction of Claudin-1 levels in the DSS group relative to healthy Control (*P* < 0.05). TM supplementation induced a gradual recovery, and the DSS + 0.75 g/kg TM and DSS + 1.5 g/kg TM high groups presented visibly stronger bands than the DSS group (*P* < 0.05). Occludin-1 showed a similar trend: DSS significantly lowered its expression (*P* < 0.05), whereas TM supplementation dose-dependently reversed this reduction. ZO-1 expression exhibited a progressive increasing pattern across the TM dosage gradient. Compared with the DSS group, the DSS + 0.75 g/kg TM and DSS + 1.5 g/kg TM groups demonstrated a pronounced restoration of ZO-1 expression (*P* < 0.05), which was highly consistent with the densitometry bar plots, further supporting the protective role of TM on epithelial barrier integrity.

### 3.6 The effect of Taraxacum on the levels of volatile fatty acid content in UC mice

**Figure 6:**
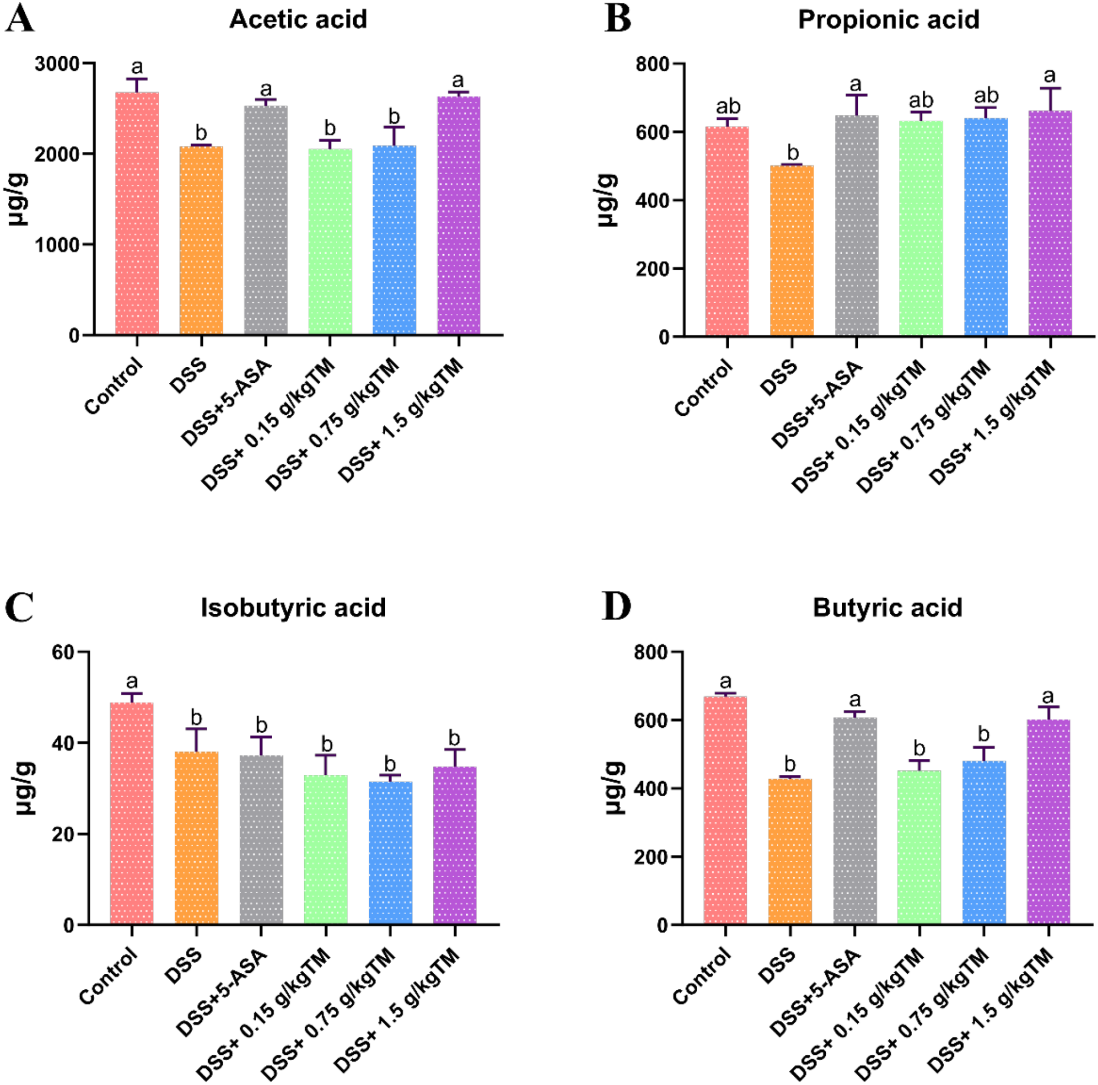
The effect of Taraxacum on volatile fatty acids in the cecum in UC mice. Bars assigned with different superscripts are significantly different (*P* < 0.05). **(A)** Acetic acid content; **(B)** Propionic acid content; **(C)** Isobutyric acid content; **(D)** Butyric acid content. The value is the mean ± SEM.

In comparison to the Control group, the DSS-treated mice demonstrated a significant reduction of acetic, propionic, isobutyric, and butyric acids in the cecal digesta (**Figure 6**; *P* < 0.05). In contrast, TM intervention successfully reinstated the levels of acetic, propionic, and butyric acids in the cecal digesta, counteracting the deficiencies noted in the DSS group (*P* < 0.05). In addition, the 1.5 g/kg dosage was the most effective regimen, producing the highest amounts of acetic, propionic, and isobutyric acids among all TM-treated groups (*P* < 0.05).

### 3.7 The effect of Taraxacum on the intestinal flora of UC mice

**Figure 7:**
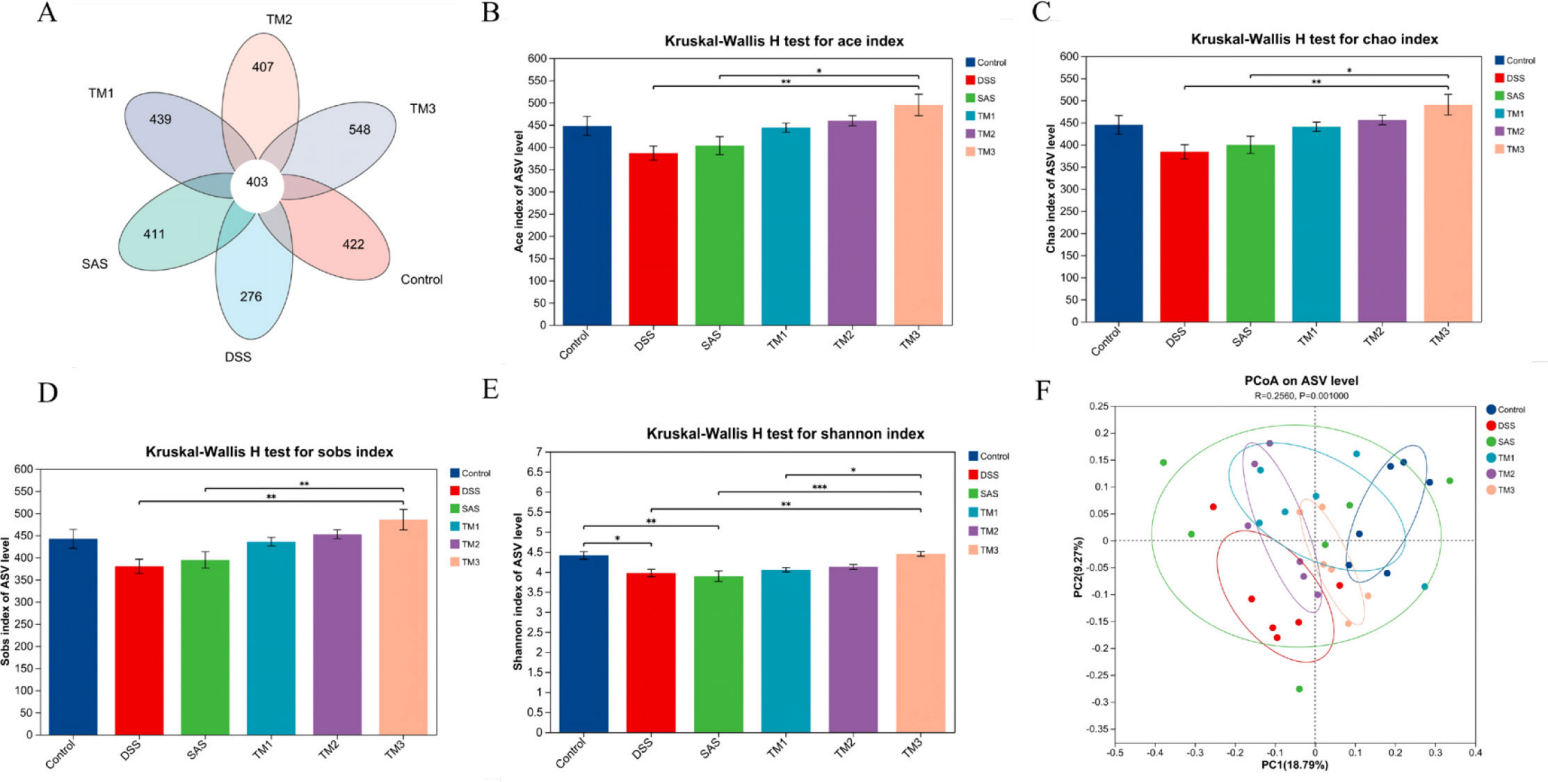
Summary of the microbial community in the intestinal contents of mice. Control (Control group), DSS (Dextran Sulfate Sodium group), 5-ASA (DSS + 5-ASA group), TM1 (DSS + 0.15g/kgTM group), TM2 ((DSS + 0.75g/kgTM group), TM3 ((DSS + 1.5g/kgTM group). The effect of Taraxacum on the species diversity of cecal microbiota in UC mice; **(A)** Quantity of ASVs; **(B-E)** α diversity evaluated by Ace, Chao, Sobs and Shannon index; **(F)** β diversity evaluated by principal coordinate analysis (PCoA). The value is the mean ± standard deviation. * Indicates a significant difference (*P* < 0.05).

The effect of TM on the species clustering and complexity of the cecal microbiota of DSS-stimulated mice is shown in **Figure 7A** total of 403 ASVs were found in the five treatment groups; the number of ASVs specific to the Control, DSS, 5-ASA, TM1 (0.15g/kg TM), TM2 (0.75g/kg TM) and TM3 (1.5 g/kg TM) groups were 422, 276, 411, 439, 407 and 548, respectively. Concerning alpha diversity, the TM3 group demonstrated a markedly richer microbial community, as indicated by higher values for the Ace, Sob, and Chao indices compared to the DSS model (*P* < 0.05), nonetheless, the recovery of microbial richness in the TM3 group was inferior to that of the 5-ASA positive control, as evidenced by significantly lower values for the Ace, Sob, and Chao indices (*P* < 0.05); the Shannon index in the DSS group was significantly lower in the DSS group than in the Control group (*P* < 0.05), and in the TM group than in the DSS group with the Shannon index. Exposure to DSS resulted in a significant reduction in the Shannon index, differentiating these mice from the Control group (*P* < 0.05), and in the TM group compared to the DSS group, the Shannon index increased with increasing dose, and Shannon index was markedly elevated in the TM3 group (*P* < 0.05). Principal Coordinates Analysis (PCoA) showed significant changes in species composition between DSS and Control, TM1 and TM3 groups and ANOSIM similarity analysis was performed, R = 0.2560, P = 0.042, in addition, principal component analysis revealed that the first and second principal components (PC1 and PC2) accounted for 18.79% and 9.27% of the total variance in the cecal microbiota, respectively.

**Figure 8:**
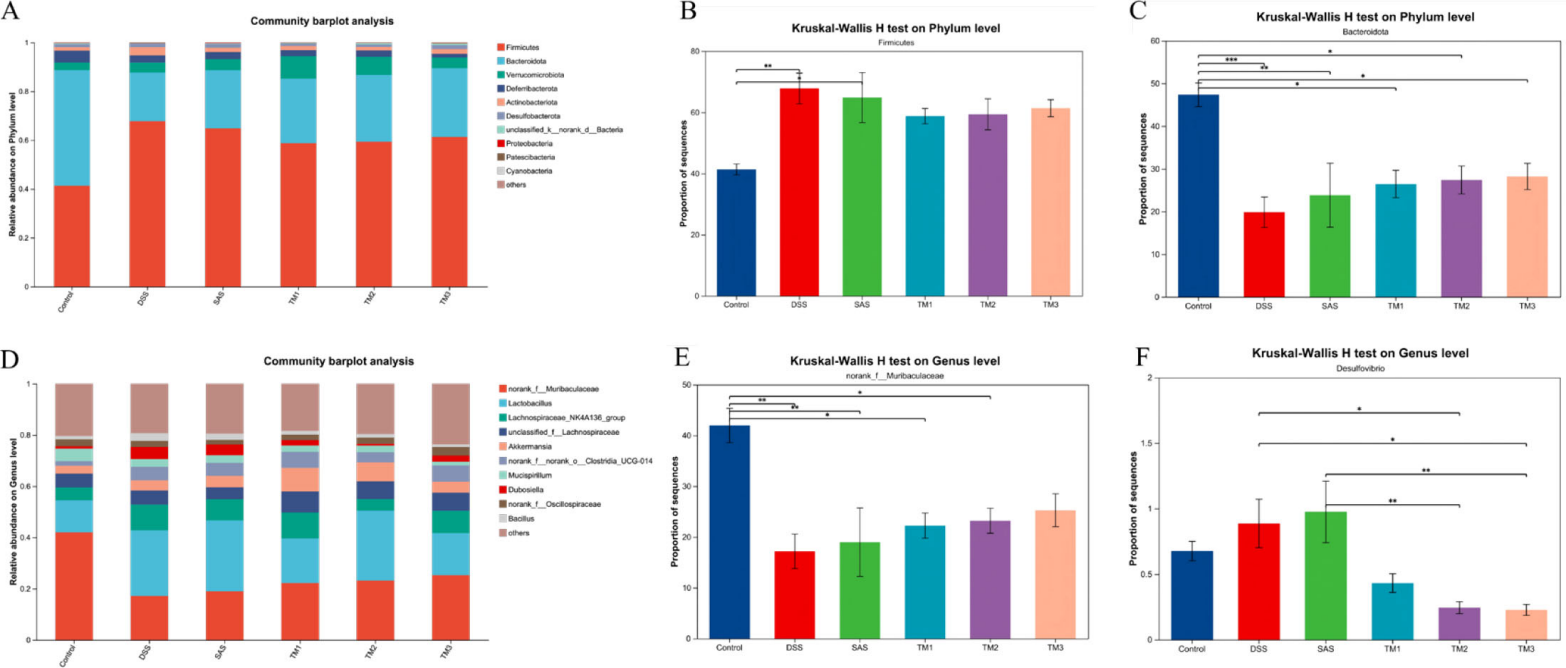
Summary of the microbial community in the intestinal contents of mice. Control (Control group), DSS (Dextran Sulfate Sodium group), 5-ASA (DSS + 5-ASA group), TM1 (DSS + 0.15g/kgTM group), TM2 (DSS + 0.75g/kgTM group), TM3 (DSS + 1.5g/kgTM group). The effect of taraxacum on the Phylum and genus levels of cecal microbiota in UC mice; **(A)** The relative abundance of the top ten bacteria at the Phylum level; The relative abundance of differential bacteria at the **(B, C)** Phylum level; **(D)** The relative abundance of the top ten bacteria at the Genus level; **(E, F)** represents the relative abundance of differentially expressed bacteria at the Genus level.

The top 10 community compositions analyzed at the species level showed that the mouse cecal microflora at the Phylum level *Firmicutes*, *Bacteroidota*, *Verrucomicrobiota*, *Desulfobacterota* and *Actinobacteriota* were the dominant Phylum (**Figure 8**). At the phylum level, the DSS group exhibited a notable increase in the relative abundance of *Firmicutes* compared to the Control group (*P* < 0.05), although the proportion of *Firmicutes* in the TM3 group was quantitatively lower than in the DSS model, this decrease was not statistically significant (*P* > 0.05). The DSS challenge resulted in a significant reduction of the *Bacteroidota* phylum in the cecal microbiota compared to the Control group (*P* < 0.05), and treatment with TM3 resulted in a slight, non-significant elevation in the proportion of *Bacteroidota* compared to the DSS group (*P* > 0.05).

The microflora of the mouse cecum was dominated by *norank_f_Muribaculaceae, Laciobacillus*, *Lachnospiraceae_NK4A136_group* and *unclassified_f:Lachnospiraceae* at the Genus level. Subsequent to DSS treatment, the relative abundance of *Norank_f_Muribaculaceae* significantly decreased, demonstrating a marked contrast to the elevated levels noted in Control (*P* < 0.05), the relative abundance of *Norank_f_Muribaculaceae* in mice within the DSS group escalated with higher doses of TM, but was not significant compared with that in the DSS group. The abundance of *Desulfovibrio* in the cecum of mice in the TM2 and TM3 groups was significantly lower compared with the DSS group (*P* < 0.05), and the relative abundance of *Desulfovibrio* in the cecum of mice in the TM2 and TM3 groups was significantly lower compared with the 5-ASA group (*P* < 0.05).

**Figure 9:**
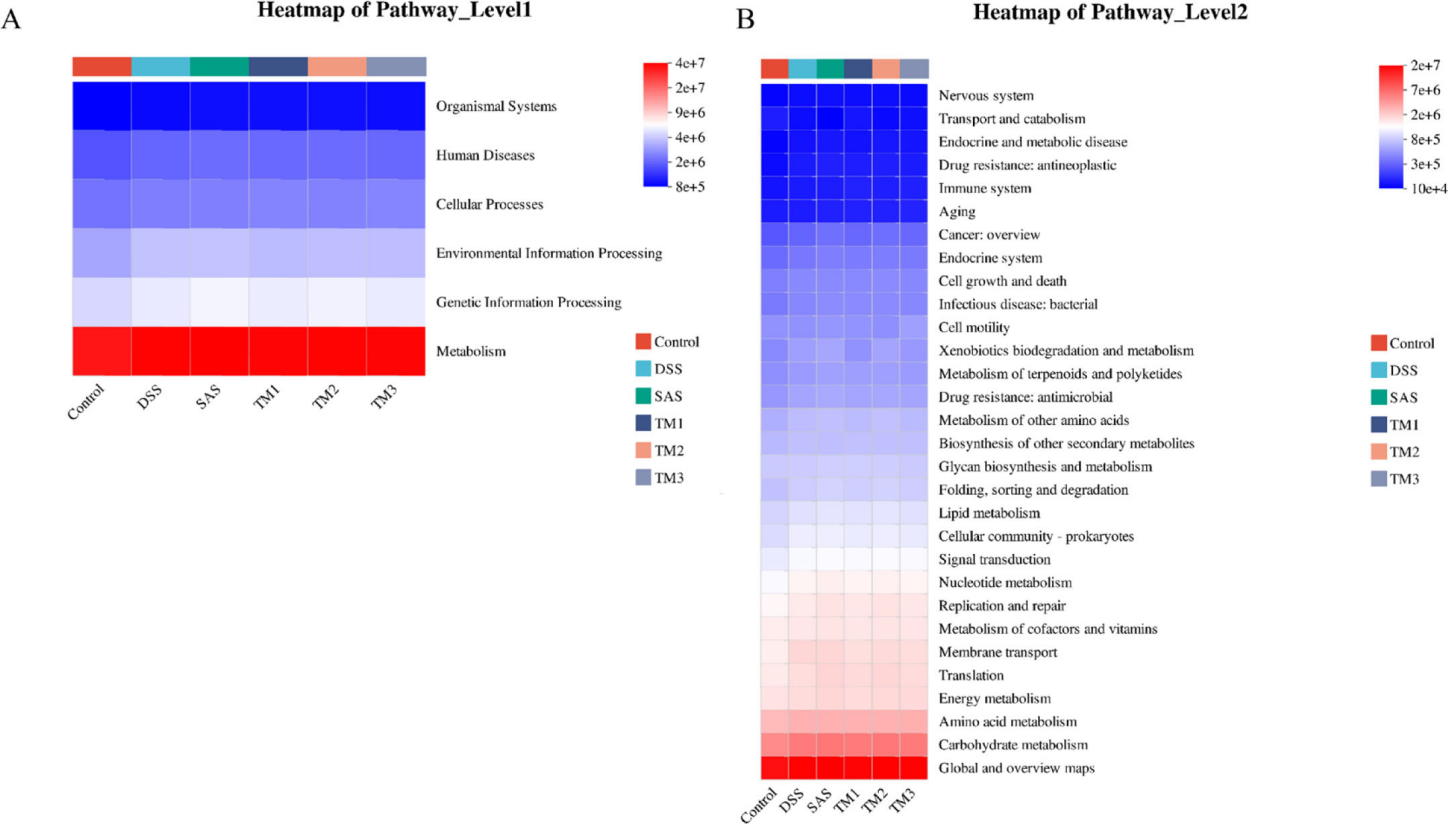
Summary of the microbial community in the intestinal contents of mice. Control (Control group), DSS (Dextran Sulfate Sodium group), 5-ASA (DSS + 5-ASA group), TM1 (DSS + 0.15g/kgTM group), TM2 (DSS + 0.75g/kgTM group), TM3 (DSS + 1.5g/kgTM group). **(A)** Heatmap of predicted KEGG pathways at Level 1 in the gut microbiota across different groups; **(B)** Heatmap of predicted KEGG pathways at Level 2 in the gut microbiota across different groups.

To further explore the functional associations between TM and the gut environment, we investigated the functional attributes of the microbiota. The KEGG pathway analysis revealed that the “Metabolism” function at Level 1 was substantially more prevalent (**Figure 9A**). At Level 2, glucose metabolism and amino acid metabolism emerged as the primary functional categories significantly influenced by the intervention (**Figure 9B**).

**Figure 10:**
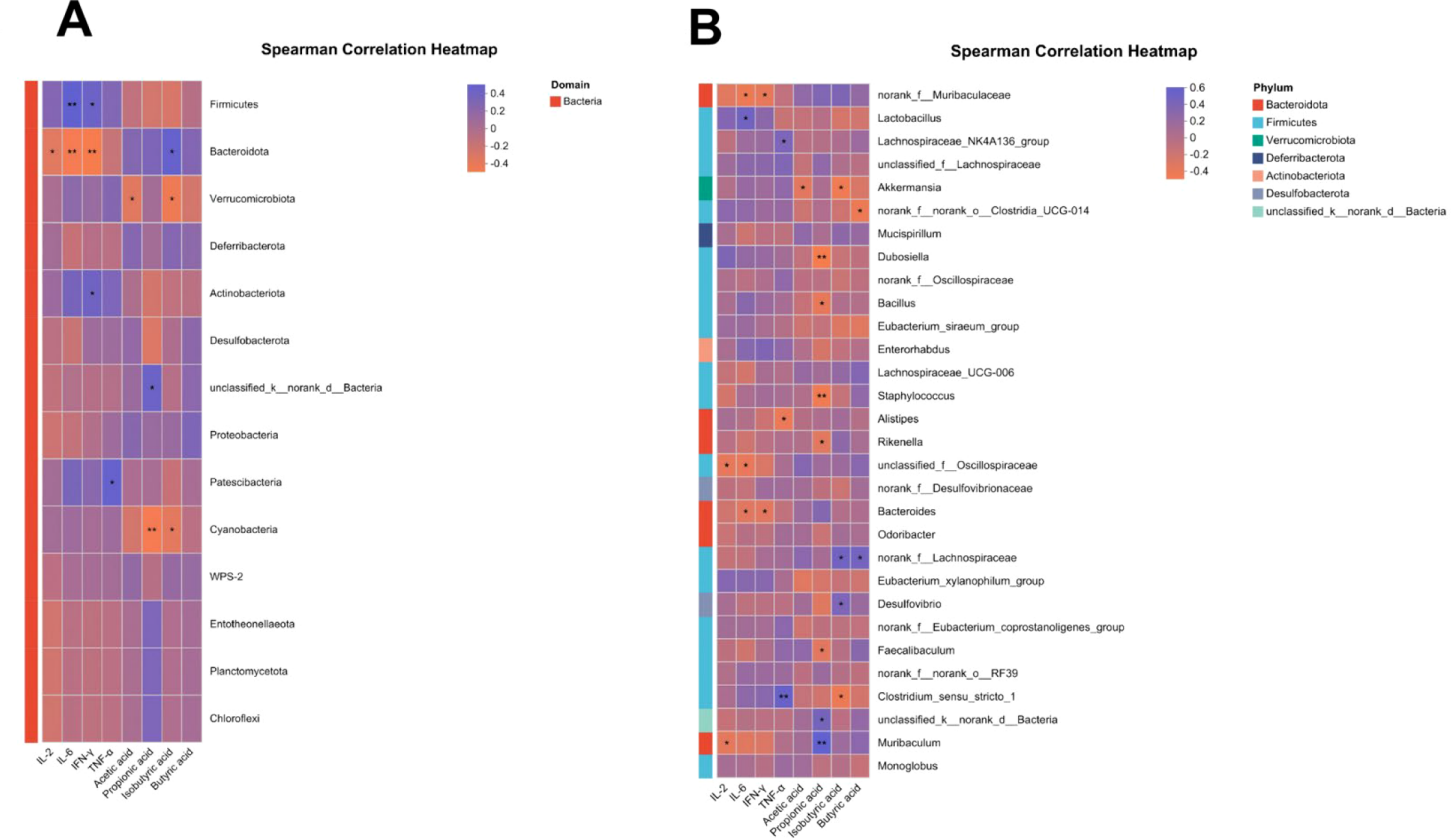
Summary of Spearman correlation heat map of microorganisms in intestinal contents of mice. Control (Control group), DSS (Dextran Sulfate Sodium group), 5-ASA (DSS + 5-ASA group), TM1 (DSS + 0.15g/kgTM group), TM2 (DSS + 0.75g/kgTM group), TM3 (DSS + 1.5g/kgTM group). **(A)** Spearman ‘s Phylum correlation analysis of intestinal contents and SCFAs immune and inflammatory indexes in mice; **(B)** Spearman ‘s Genus correlation analysis of intestinal contents and SCFAs immune and inflammatory indexes in mice.

The correlation analysis results of microbial community in intestinal contents of mice with serum inflammatory markers and VFAs are shown in **Figure 10**. Analysis showed that at the Phylum level, *Firmicutes* had a positive correlation with serum levels of IL-6 and IFN-γ in mice (*P* < 0.05). *Bacteroidota* was significantly associated with the increase of VFAs content in intestinal contents of mice (*P* < 0.05). At the genus level, the beneficial genus *Muribaculaceae*, which was reinstated by TM treatment, had a significant negative connection with the pro-inflammatory cytokines IL-6 and IFN-γ (*P* < 0.05). In contrast, the prevalence of the pathobiont *Desulfovibrio* was negatively associated with the levels of butyric acid (*P* < 0.05). These results align with the previously referenced content.

## 4 Discussion

Generally, inflammation in IBD occurs in the colonic mucosa and submucosa (Kang et al., 2022). This study demonstrated for the first time that TM can alleviate colonic inflammation in DSS-induced IBD mice at both macroscopic and molecular levels. In animal models, the colitis model induced by DSS had similar clinical symptoms to human UC. The administration of DSS compromised the integrity of tight junctions among intestinal epithelial cells, consequently worsening intestinal permeability, and allowed translocation of bacteria and endotoxins (Zhou et al., 2024). After exposure to intestinal lumen antigens, inflammatory cells such as neutrophils and macrophages are recruited, leading to the secretion pro-inflammatory factors such as TNF-α and IL-6 (Dharmani and Chadee, 2008). Therefore, in this study, we used 3% DSS to establish the UC mouse model.

Research has shown that DSS-induced IBD mice had colon shortening, immediate mucosal damage, infiltration of inflammatory cells, crypt destruction, and an elevated histology score (Zhang et al., 2020). In our current analysis, we observed that DSS-induced mice presented symptoms such as weight loss, reduced colon length, and elevated DAI score. Both TM administration and positive 5-ASA intervention alleviated weight loss and increased DAI score induced by DSS. Histopathological examination revealed that colonic tissues from the DSS group displayed significant pathological changes, marked by extensive lymphocyte infiltration, congestion, edema, and mucosal ulceration. Significantly, in mice administered TM and 5-ASA, he severity of inflammatory cell infiltration and crypt structural damage was markedly attenuated This result aligns with that of Li et al (Li et al., 2022). TM intervention may impede weight loss, reduce DAI score, avert colonic length reduction, and fortify the colonic mucosal architecture.

Oxidative stress may impair the architecture of the intestinal mucosa, augment intestinal permeability, and contribute to inflammatory bowel disease inflammatory bowel diseases (such as Crohn’s disease and ulcerative colitis) (Grisham, 1994). Multiple enzymes, such as SOD, CAT, and GPx, are essential in mitigating reactive oxygen species and diminishing oxidative damage within the antioxidant system (Chen Y. P. et al., 2021). MDA is the primary oxidation product of polyunsaturated fatty acids and serves as a significant marker of oxidative damage to membrane lipids (Valko et al., 2007). It has been noted that the hydroalcoholic extract of Taraxacum protects liposomes from lipid peroxidation, scavenges free radicals, and exhibits significant antioxidant activity (Yan et al., 2024). In this experiment, TM decreased the elevated MDA levels induced by DSS in mice. Li et al (Li et al., 2024). found that Taraxacum polysaccharides restored the expression levels of CAT, Nrf2, HO-1 and NQO1 genes to normal levels to a certain extent, which alleviated oxidative stress *in vivo* and was beneficial to the animal health. The findings of this experimental investigation indicated that 1.5 g/kg TM reduced the DSS stress-induced decrease in serum T-AOC, GPX, SOD, and CAT levels in mice, indicating that TM was able to alleviate DSS-induced oxidative stress in mice. Choi et al (Choi et al., 2010). research showed that Taraxacum officinale increased GSH-Px and CAT content and improved liver health in rabbit liver tissue. Meanwhile, Taraxacum officinale polysaccharide showed good performance in DPPH radical scavenging efficacy and hydroxyl radical scavenging capability (Cai et al., 2019). Consequently, Taraxacum may enhance the body’s antioxidant capacity and mitigate cellular oxidative damage, suggesting its potential application in the treatment of ulcerative colitis.

Cytokine secretion is essential for initiating host innate defense mechanisms and controlling adaptive immune responses. TM also alleviates the inflammatory response induced by DSS stress by modulating cytokine levels in the colon. Administration of DSS in the drinking water of mice often leads to severe inflammatory responses (Hsu et al., 2020). Pro-inflammatory cytokines, including IL-2, IL-6, IFN-γ, and TNF-α, are recognized for their critical role in undermining intestinal barrier integrity and worsening mucosal permeability (Al-Sadi et al., 2009; Zhang H. et al., 2022). The current investigation demonstrated that levels of IL-2, IL-6, IFN-γ, and TNF-α were raised in mice consuming DSS, validating the successful creation of the ulcerative colitis model in this experimental group. However, current treatments for ulcerative colitis have undesirable side effects. Taraxacum, a natural and safe ingredient, has excellent anti-inflammatory properties (Wang et al., 2021). Building on these properties, our study evaluated its therapeutic potential. As anticipated, we found that treatment with TM significantly attenuated the elevated levels of IL-2, IL-6, IFN-γ, and TNF-α in the colons of mice. Consistent with the present study, previous studies have shown that an aqueous extract of Taraxacum leaves reduced serum levels of IFN-γ, TNF-α, IL-4, and IL-10 in mice infected with *Schistosoma haematobium*, as well as reducing the number of CD4^+^ and CD25^+^ cells in the blood and spleen, and inhibiting alterations in apoptosis-associated gene expression in the spleen of mice (El-Refaiy et al., 2025). Furthermore, studies demonstrate that Taraxacum modulates the release of inflammatory cytokines within the gastrointestinal tract, an effect closely linked to all immune function (Tan et al., 2018). Pro-inflammatory cytokines, including IL-2, IL-6, IFN-γ, and TNF-α, are recognized for their critical role in undermining intestinal barrier integrity and worsening mucosal permeability (Che et al., 2019). In our study, the considerable effectiveness of TM in inhibiting inflammatory reactions was observed concurrently with a suppressed synthesis of inflammatory cytokines. This finding provides a potential strategy for intervention in immune-related pathological states. In conclusion, TM and its bioactive constituents significantly reinstated immunological homeostasis in mice following TM treatment is strongly associated with the diminished the overexpression of critical inflammatory mediators.

VFAs as the principal metabolites of fermented polysaccharides in the microbiota, participate in several metabolic processes of the host, including energy use, intestinal anti-inflammation, and intestinal motility (Wan et al., 2018; Duscha et al., 2022). Research indicates that butyric acid serves as the primary energy source for intestinal cells, supplying over 70% of their energy and facilitating the growth of intestinal epithelial cells (Zhang K. et al., 2022). Prior research indicates that the administration of Achillea L effectively protects the stomach mucosa of rats by increasing acetic acid production (Saeidnia et al., 2011). Researchers (Wei et al., 2025) found that Angelica dahurica polysaccharides alleviate colitis in mice by regulating intestinal microbiota and SCFA metabolism. Certain investigations have shown that bovine polysaccharides ameliorated CTX-induced dysbiosis of the intestinal microbiota and enhanced the synthesis of volatile fatty acids in mice (Chen X. et al., 2021). This study parallels the aforementioned research findings. Taraxacum enhanced the levels of acetic acid, propionic acid, and butyric acid in the cecal contents of DSS-induced mice, These favorable metabolic shifts tightly correlate with the restoration of intestinal health observed in our model.

The mammalian gastrointestinal tract is populated by trillions of microorganisms, which have evolved in concert with the host over a long period of time to form a mutually beneficial symbiotic ecosystem, a multi-level symbiotic defense system that not only protects the host from pathogen invasion, but also influences physiological functions such as nutrient absorption and energy metabolism in the host by preserving the equilibrium of the gut microbiota (Quin and Gibson, 2020). Numerous studies use Chao and Ace indices to assess species abundance, while Simpson and Shannon indices are employed to evaluate species diversity (Wang et al., 2022). The 1.5g/kg TM group exhibited markedly elevated Ace, Chao, Sob, and Shannon indices relative to the DSS group, indicating an enhancement in the alpha diversity of the gastrointestinal microbiota. The results of the present study revealed a significant increase in the relative abundance of *Bacteroidota* in the cecum of mice in the TM group. Previous studies have shown that *Bacteroidota* produces metabolites such as SCFAs through the breakdown of dietary fiber. These SCFAs provide energy to intestinal epithelial cells, promotes the expression of tight junction proteins, strengthens the intestinal barrier, and reduces the entry of harmful substances into the bloodstream (Amabebe et al., 2020; Liu et al., 2020). Certain species of the *Bacteroidota* phylum, particularly *Bacteroides fragilis*, are recognized for facilitating the development of regulatory T cells, hence mitigating dysregulated Th1/Th2 immunological responses, and safeguarding mice against pathogen-induced colitis (Mazmanian et al., 2008). This aligns with our previous immunological and VFA data. Conversely, certain members of the *Firmicutes* have been implicated in the development of intestinal and systemic diseases such as inflammatory bowel disease and intestinal injuries (Sokol et al., 2008; Frank et al., 2011), for example, a member of the *Firmicutes*, *segmented filamentous bacteria*, has been identified as a promoter of the development and proliferation of Th17 cells in the intestinal tract and contribute to the inflammation seen in ulcerative colitis in adults (Ivanov and Littman, 2010; Gálvez, 2014). In the current investigation, DSS activation at the Phylum level increased the prevalence of *Firmicutes*, maybe linked to intestinal inflammation, whereas The TM therapy mitigated these phenomena and increased the prevalence of *Bacteroidota*. These data indicate that the therapeutic effects of TM are closely associated with a modulated gut microbiota profile, particularly an enhanced *Bacteroidota* to *Firmicutes* ratio, which parallels the alleviation of DSS-induced ulcerative colitis.

Members of the family *Muribaculaceae* may produce volatile fatty acids (VFAs) by metabolizing complex carbohydrates such as dietary fiber. These VFAs not only provide an energy source for intestinal epithelial cells but also stimulate the production of tight junction proteins, which are effective in defending against pathogen invasion and reducing ectopic spread of endotoxin (Smith et al., 2019). This might be associated with the fact that *Muribaculaceae* are a mucin-degrading family that maintaining the integrity of the mucosal barrier. These features safeguard intestinal epithelial cells from mechanical stress and exert an immunological impact to promote intestinal homeostasis (Pan et al., 2022).To assess the homeostasis of the gut commensal microbiota, we analyzed the relative abundance of the *norank_f:Muribaculaceae* (Genus level; **Figure 8E**). As a core dominant bacterial group in the murine gut, *Muribaculaceae* maintained a high abundance in the Control group. Moreover, DSS treatment led to a significant reduction in its abundance vs. Control (*P* < 0.05), indicating that colitis severely disrupted the native intestinal microecological structure. IBD significantly reduces the prevalence of *Muribaculaceae* in the gut microbiota, aligning with our experimental findings. Furthermore, *Muribaculaceae* competes with pathogens for ecological niches and nutrients within the intestinal mucus layer, thereby resisting colonization by intestinal pathogens to preserve microbial homeostasis (Pereira et al., 2020). Although the abundance of *Muribaculaceae* in the 5-ASA group did not fully recover to normal levels, intervention with TM extract exhibited a dose-dependent recovery trend. As the TM dosage increased, the relative abundance of *Muribaculaceae* gradually rebounded. Consequently, we hypothesize that the mitigation of DSS-induced colonic inflammation in mice after TM treatment is intricately linked to the modulation of gut microbiota, particularly the enhancement of the beneficial *Muribaculaceae* and the simultaneous decrease of the pathobiont *Desulfovibrio*, but the specific molecular mechanisms of these gut microbes still need to be further explored.

*Desulfovibrio* is classified as a sulfate-reducing bacterium capable of converting sulfate into the hazardous chemical hydrogen sulfide (H_2_S), which can induce oxidative stress at high concentrations, disrupting the antioxidant defense system of intestinal epithelial cells, leading to the accumulation of reactive oxygen species, and exacerbating tissue damage (Yang et al., 2016). Simultaneously, H_2_S may directly harm intestinal epithelial cells and impair intestinal barrier function, resulting in increased intestinal permeability and inciting chronic inflammation. An increased abundance of *Desulfovibrio* has been linked to the probability of recurrence of inflammatory bowel illnesses, including ulcerative colitis and Crohn’s disease (Rowan et al., 2010; Rohr et al., 2020). At the genus level, we analyzed the relative abundance of *Desulfovibrio* in the gut microbiota of mice across all groups. As shown in the **Figure 8**, compared with the Control group, the abundance of *Desulfovibrio* in the DSS model group showed an upward trend, indicating that colitis induction was accompanied by an enrichment of this harmful bacterium. However, intervention with Taraxacum extract (TM) significantly reversed this trend. Specifically, compared with the DSS group, the relative abundance of *Desulfovibrio* in the TM1 and TM2 groups was significantly reduced (*P* < 0.05), suggesting that the extract effectively inhibited the overgrowth of this bacterium. Notably, the suppressive effect of the TM groups was superior to that of the positive control drug, 5-ASA. Statistical analysis revealed that the abundance of *Desulfovibrio* in the TM2 and TM3 groups was significantly lower than that in the 5-ASA group (*P* < 0.05). KEGG analysis found that TM may possess unique advantages in reducing hydrogen sulfide-producing bacteria and restoring intestinal microecological balance. This conclusion closely corresponds with recent studies that illustrate the crucial role of microbial metabolites in maintaining gut health. The fermentation of dietary fiber and the subsequent generation of VFAs are often linked to improved glucose metabolism. VFAs are crucial for maintaining intestinal barrier integrity as they enhance the functionality of proteins that form tight junctions. They also confer significant power to the cells constituting the colon. Alterations in bacterial metabolism of amino acids, particularly tryptophan, can produce specific metabolites that regulate inflammation in the mucosa. These functional modifications indicate that Taraxacum may assist in colitis by altering the composition of the microbial community and inducing the microbiota into a protective metabolic state that accelerates the synthesis of VFAs and the restoration of the barrier.

Currently, our research cannot ascertain whether TM efficacy arises from a direct effect on intestinal tissues or from indirect modulation through microbial metabolites. Future research utilizing germ-free animals, antibiotic depletion, and *in vitro* experiments is crucial to comprehensively clarify these unique pathways.

In conclusion, TM may impede the reduction of colon length in UC mice, modulate the secretion of inflammatory mediators and oxidative stress levels. The treatment efficacy is intricately linked to the prevention of colon shortening and the regulation of inflammatory mediator release and oxidative stress levels. Moreover, the improvement of colitis after TM management is associated with the reinstatement of gut microbiota richness and variety. By correcting DSS-induced dysbiosis—marked by the proliferation of advantageous commensals and the suppression of pathobionts—TM is essential for sustaining normal colonic function and ensuring intestinal homeostasis.

## Data Availability

The original contributions presented in the study are included in the article/**Supplementary Material**. Further inquiries can be directed to the corresponding authors.
