# Supplementary figures and images for: Taraxacum alleviates ulcerative colitis, accompanied by the modulation of gut microbiota and restoration of intestinal barrier integrity
Source: Front Cell Infect Microbiol. 2026 Mar 27;16:1778487. doi: 10.3389/fcimb.2026.1778487 (PMC13066173; doi:10.3389/fcimb.2026.1778487)

Supplementary Information

A full scan of the entire original gel(s)





Actin


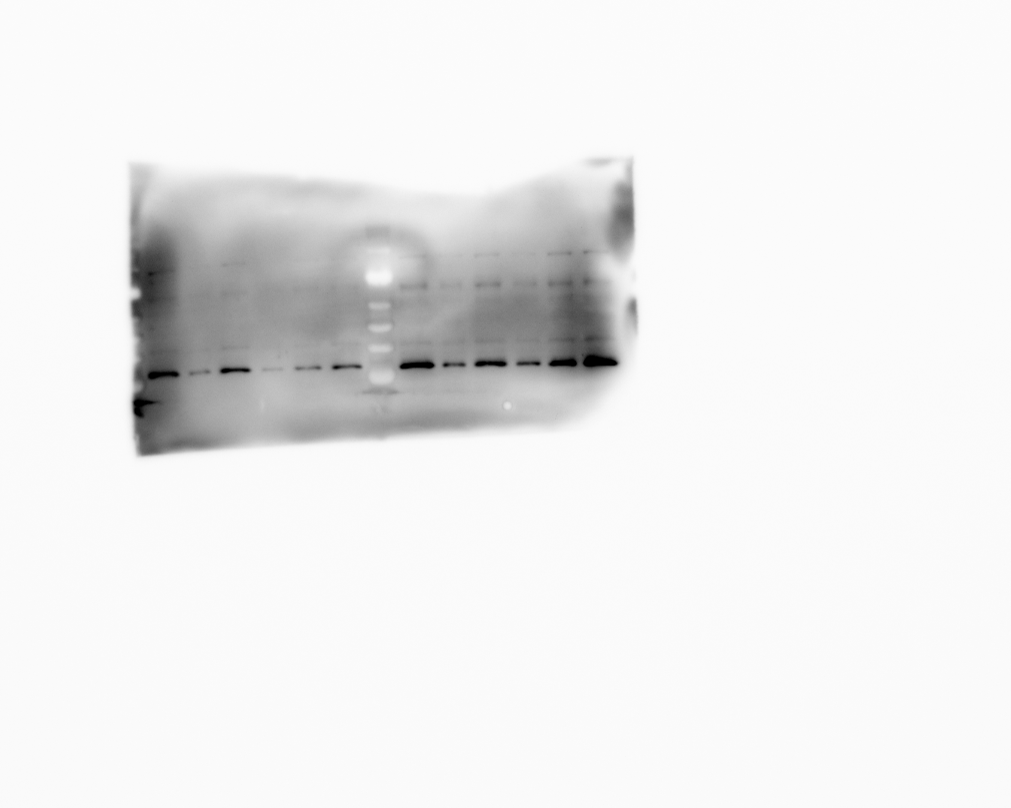


Claudin-1


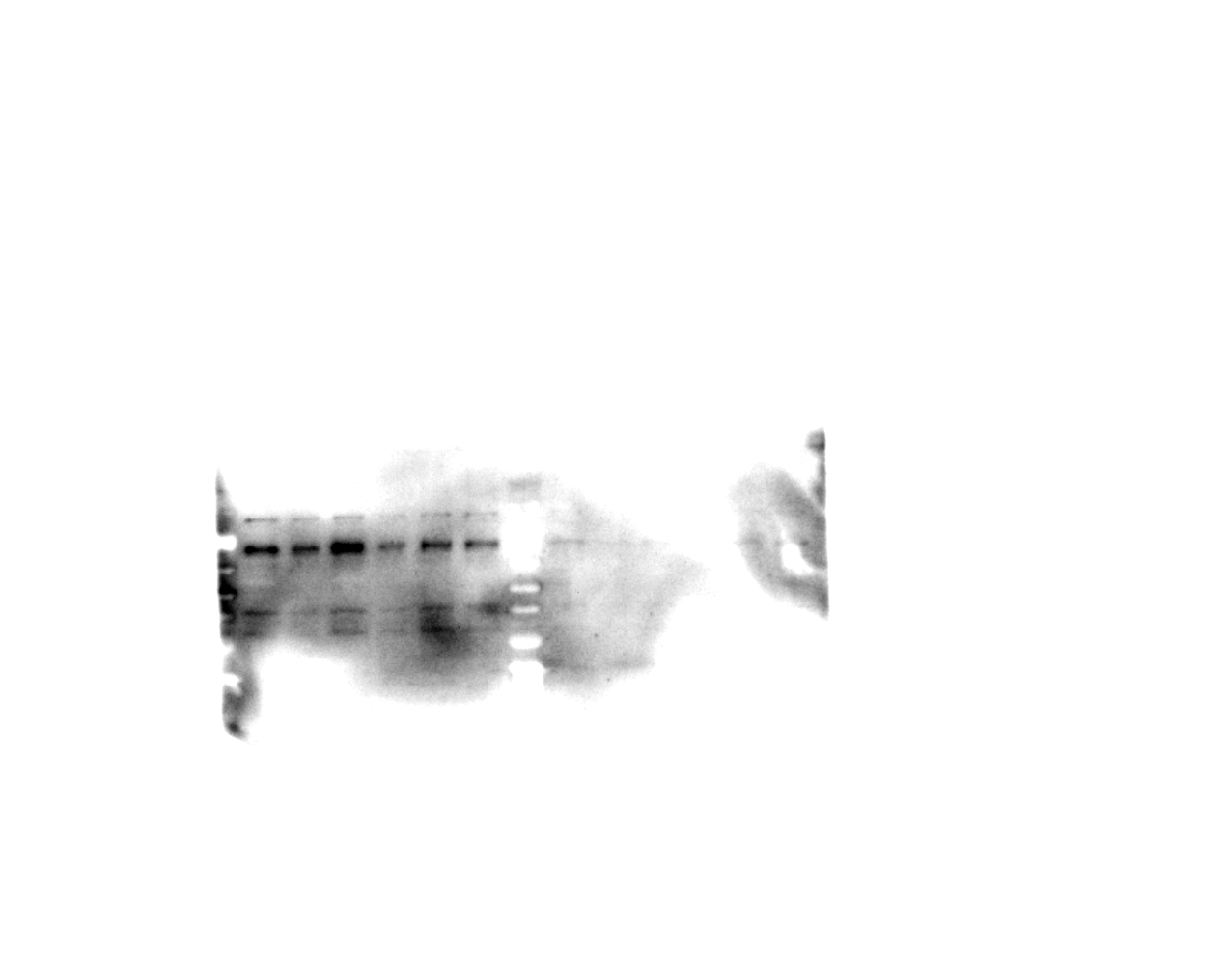


Occludin-1


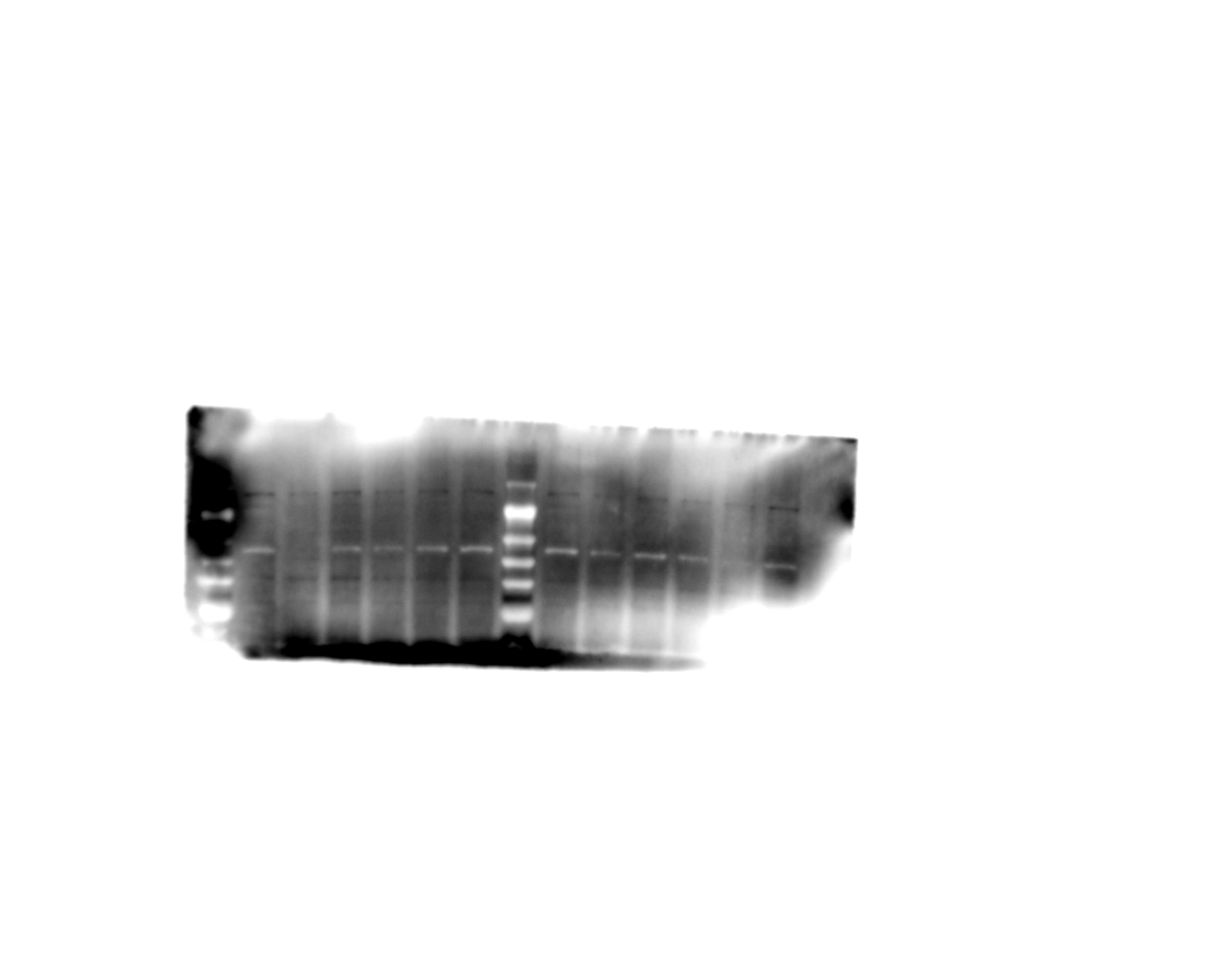


Zo-1

Supplement: Supplementary file 1 [file Table1.docx]
